# Prevalence of Thermophilic *Campylobacter* in Cattle Production at Slaughterhouse Level in France and Link Between *C. jejuni* Bovine Strains and Campylobacteriosis

**DOI:** 10.3389/fmicb.2018.00471

**Published:** 2018-03-19

**Authors:** Amandine Thépault, Typhaine Poezevara, Ségolène Quesne, Valérie Rose, Marianne Chemaly, Katell Rivoal

**Affiliations:** ^1^ANSES, Hygiene and Quality of Poultry and Pig Products Unit, Ploufragan-Plouzané Laboratory, Bretagne-Loire University, Ploufragan, France; ^2^UFR of Life Sciences and Environment, University of Rennes 1, Rennes, France

**Keywords:** *Campylobacter jejuni*, MLST, CGF40, bovine, foodborne disease

## Abstract

*Campylobacter* is the leading cause of bacterial gastroenteritis in industrialized countries, with poultry reservoir as the main source of infection. Nevertheless, a recent study on source attribution showed that cattle could be a source of human contamination in France (Thépault et al., 2017). However, few data are available on thermophilic *Campylobacter* epidemiology in cattle in France. The aim of this study is to collect new data of thermophilic *Campylobacter* prevalence in these animals and to subtype *C. jejuni* isolates to assess the potential implication of cattle in campylobacteriosis. A 6-month survey was carried out in one of the largest European slaughterhouse of cattle. Based on a statistical representative sampling plan, 959 intestinal content samples (483 adult cattle and 476 calves) were collected. An adapted version of the ISO 10272 standard and Maldi-Tof were used for detection and speciation of thermophilic *Campylobacter* isolates. Within more than 2000 thermophilic *Campylobacter* isolates collected, a selection of 649 *C. jejuni* isolates was typed with Comparative Genomic Fingerprinting (CGF40) and a subset of 77 isolates was typed using Multilocus Sequence Typing (MLST). Simultaneously, clinical isolates occurred in France were genotyped. Prevalence of thermophilic *Campylobacter* in the global cattle population was 69.1% (CI_95%_ = 66.1, 72.1) at slaughterhouse level. In adult cattle, the prevalence was 39.3%, while 99.4% of calves were contaminated, and *C. jejuni* was the most prevalent species with prevalence of 37.3 and 98.5%, respectively and a higher genetic diversity in adult cattle. The prevalence of *C. coli* was lower with 3% in adult cattle and 12.5% in calves. MLST and CGF40 genotyping did not showed a high number of clusters within cattle isolates but the predominance of few clusters accounted for a large part of the population (CC-21, CC-61, CC-48, and CC-257). By comparison with clinical genotypes, genetic diversity was significantly lower in cattle. Moreover, significant overlap was observed between genotypes from both origins, with 3 of the 4 main cattle clusters present in human isolates. This study provides new insights on the epidemiology of thermophilic *Campylobacter* and *C. jejuni* in cattle production in France and their potential implication in human infection.

## Introduction

Thermophilic *Campylobacter*, especially *C. jejuni* and *C. coli*, are the primary cause of human bacterial gastroenteritis worldwide and are major zoonotic agents. In Europe, the number of reported confirmed cases of human campylobacteriosis was 229,213 with an European notification rate of 65.6 per 100,000 population in 2015 (EFSA and ECDC, 2016). However, these data are often an underestimate of the true incidence (Havelaar et al., 2013). Thus, in France a recent study estimated that more than 500,000 cases of campylobacteriosis occur each year (Van Cauteren et al., 2015), while EFSA and ECDC (2016) reported 6,000 cases in 2015. In France, *C. jejuni* is responsible for nearly 80% of human infections while *C. coli* accounted for 15% of campylobacteriosis between 2003 and 2010 (Bessède et al., 2014).

While poultry products are well-recognized sources of human infection, there is increasing evidence that ruminants also play a prominent role (Mughini Gras et al., 2012; Fernández et al., 2015). A recent study performed in France (Thépault et al., 2017), estimated that equal proportions of clinical isolates could be attributed to chicken and ruminant reservoirs, using 15 novel host-segregating markers for source attribution, defined on a gene-by-gene approach (Sheppard et al., 2012) based on a pan-genomic approach (Méric et al., 2014). In the same way, de Haan et al. (2010) showed that cases of human *Campylobacter* infections in Finland could be attributed equally to cattle and poultry using MLST data. Moreover, another study from the United-Kingdom using MLST genotype data, estimated that 42% of clinical cases from young children in rural areas arose via acquisition of *C. jejuni* from cattle sources and 12% from sheep sources (Wilson et al., 2008; Strachan et al., 2012). Nevertheless, this finding is not surprising since several studies reported that the cattle reservoir seems to be highly contaminated. Indeed, in the USA, a recent study described a prevalence of *Campylobacter* about 71% at three cattle farms (Cha et al., 2017). In Finland, the prevalence of *Campylobacter* spp. in cattle was evaluated at 31.1% in a 12-months sampling survey (Hakkinen et al., 2007), while it reached 54.6% in the United Kingdom (Milnes et al., 2008) and 22% in Scotland (Rotariu et al., 2009). Therefore, it is important to explore the epidemiology of cattle contamination by *Campylobacter* in order to explain the role of this animal reservoir in human infections. Actually, few data are available in France, since only one study has been conducted between 2002 and 2006, which showed a prevalence of thermophilic *Campylobacter* spp. of 4.6% in cull cows, 6% in young cattle, and 39.1% in calves (Châtre et al., 2010). However, relationships between thermophilic *Campylobacter* isolates from human and cattle were not investigated in this study. Therefore, the role of cattle in human contamination with thermophilic *Campylobacter* in France remains unknown.

In order to investigate the epidemiology of thermophilic *Campylobacter* spp., molecular subtyping methods with enhanced discriminatory power are required. In recent years the Comparative Genomic Fingerprinting (CGF) technique has been used to subtype *C. jejuni* and *C. coli* (Taboada et al., 2012; Deckert et al., 2014). This technique represents a high resolution subtyping approach which assesses genetic variability in the accessory genome. Moreover this technique is complementary to Multilocus Sequence Typing (MLST) which remains the gold standard for *Campylobacter* genotyping (Dingle et al., 2001).

The purpose of this work was to conduct a representative investigation in the cattle production in France to estimate the prevalence of thermophilic *Campylobacter* spp. in this reservoir and characterize further *C. jejuni* isolates using CGF and MLST. Then, the genotypes from cattle isolates were compared to genotypes found in isolates from clinical cases occurred in 2009 and 2015 in France to assess the potential role of cattle in human campylobacteriosis.

## Materials and methods

### Sampling plan

A 6-month survey was carried out in one of the largest European slaughterhouse of cattle from June to December 2016. The number of intestinal contents to be sampled was calculated in relation to the number of slaughtered animals in the abattoir (60 000 calves and 140 000 adults bovines slaughtered each year), with an expected animal-prevalence of 16.5% (Châtre et al., 2010) and a precision of 20% with 95% confidence limits (Machin et al., 2009). Thus, the representative sampling plan consisted of sampling 959 intestinal contents taken from 483 adult cattle and 476 calves. The 959 samples were from 282 farms distributed among 32 French departments representative of the French production of cattle. Among the adult cattle (>8 months old), 270 animals were from beef breed and 213 from dairy breed, while among calves 143 were beef calves, 328 dairy calves, two animals belonged to mixed breed and breed information was missing for three calves (Table 1).The samples were collected weekly during 6 months at the evisceration level in the abattoir, transported to the laboratory in isotherm bag and stored at + 4°C until the analysis.

**Table 1:** Prevalence of *Campylobacter* species in adult cattle and calves in France.

	**Adult cattle**									**Calves**												
	**Beef (*****n*** = **270)**			**Dairy (*****n*** = **213)**			**Total (*****n*** = **483)**			**Beef (*****n*** = **143)**			**Dairy (*****n*** = **328)**			**Mixed (*****n*** = **2)**		**Unknown (*****n*** = **3)**		**Total (*****n*** = **476)**		
	***n***	**%**	**95% CI**	***n***	**%**	**95% CI**	***n***	**%**	**95% CI**	***n***	**%**	**95% CI**	***n***	**%**	**95% CI**	***n***	**%**	***n***	**%**	***n***	**%**	**95% CI**
*Campylobacter* spp.	*90*	33.3^**^	27.7–39.3	*100*	46.9^**^	40.1–53.9	*190*	39.3^*^	34.9–43.9	*142*	99.3	96.2–99.9	*326*	99.4	97.8–99.9	*2*	100	*3*	100	*473*	99.4^*^	98.2–99.9
*C. jejuni*	*85*	31.5^***^	26.0–37.4	*95*	44.6^***^	37.8–51.6	*180*	37.3	32.9–41.8	*142*	99.3	96.2–99.9	*322*	98.2	96.1–99.3	*2*	100	*3*	100	*469*	98.5	96.9–99.4
*C. coli*	*7*	2.6	1.1–5.3	*7*	3.3	1.3–6.7	*14*	2.9	1.6–4.8	*20*	14.0	8.8–20.8	*39*	11.9	8.6–15.9	0	0	0	0	*59*	12.4	9.6–15.7
*C. lari*	*0*	0		*1*	0.5	0.01–2.6	*1*	0.2	0.005–1.15	*0*	0		0	0		*0*	0	0	0	*0*	0	
*C. fetus*	*1*	0.4		*0*	0		*1*	0.2	0.005–1.15	*0*	0		0	0		*0*	0	0	0	*0*	0	
*C. hyointestinalis*	*0*	0		*0*	0		*0*	0		*1*	0.7	0.02–3.8	*0*	0		*0*	0	0	0	*1*	0.2	0.005–1.15

*The mention “mixed” indicates a mixed breed. The mention “unknown” indicates the absence of information about the breed of the animal*.

* *Indicates a significant difference between prevalence scores based on chi-square test (p < 0.001)*.

** *Indicates a significant difference between prevalence scores based on chi-square test (p < 0.001)*.

*** *Indicates a significant difference between prevalence scores based on chi-square test (p < 0.001)*.

### Isolation and identification of thermophilic *Campylobacter* species

Isolation of thermophilic *Campylobacter* spp. was carried out 1 day after the sample collection according to an adapted version of the ISO standard 10272. Direct plating of one loop of intestinal content was performed on two media mCCDA (modified Charcoal Cefoperazone Deoxycholate Agar) and Butzler n°2 (Oxoid, Dardilly, France). The agar plates were incubated during 72 ± 2 h. In addition, ten grams of intestinal content were added to 90 ml of Preston broth for enrichment. After incubation during 24 ± 2 h, streaking of the broths was performed on mCCDA and Butzler n°2 plates whose were incubated during 48 h. All incubations of broths and plates were done in microaerophilic atmosphere (85% N_2_, 10% CO_2_, 5% O_2_) at 41.5°C. All the agar media were examined to detect the presence of thermophilic *Campylobacter* typical colonies. For each positive sample, one colony with thermophilic *Campylobacter* characteristics (morphology and mobility) per plate (two different media, two conditions for the detection: direct or after enrichment) were subcultured on blood agar. Thus, one to four isolates were collected from each positive sample leading to a collection of 2226 thermophilic *Campylobacter* isolates. The species of all the collected isolates were identified using MALDI-TOF MS (Bessède et al., 2011). The isolates were stored at −70°C in peptone broth containing 20% (v/v) glycerol and only *C. jejuni* isolates were characterized further in this study.

### Clinical isolates

Clinical isolates were obtained from the National Reference Center for *Campylobacter* and *Helicobacter* in France. These isolates were collected from campylobacteriosis cases which occurred in 2009 (*n* = 143) (Thépault et al., under review) and 2015 (*n* = 371) (Rose et al., 2017) in the 10 most populated departments in France.

### CGF40 typing of *Campylobacter jejuni*

A selection of 649 isolates of *C. jejuni* (one isolate per positive animal) was genotyped using the CGF typing method based on 40 assay genes (CGF40). This method was used to genotype a high number of isolates with a higher discriminatory power than MLST at lower cost and with highly concordant results (Taboada et al., 2012). Isolates were subcultured onto *Campylobacter* blood-free selective agar® (Karmali, Oxoid) in microaerobic conditions at 41.5°C for 48 h. The genomic DNA was extracted from 1-day single-colony cultures using the InstaGene® Matrix (BioRad Laboratories, Hercules, CA, USA) according to the manufacturer's instructions and quantified using a NanoDrop® ND-1000 (Thermo Scientific, Gometz le Châtel, France).

The experimental conditions to generate CGF fingerprints were adapted from the original protocol described by Taboada et al. (2012). The experimental details for the amplification of the 40 assay genes were previously published (Thépault et al., 2016). The CGF fingerprints were then visualized using a standard gel electrophoresis containing 2% of agarose colored with GelRed® (Interchim, Montluçon, France) in accordance with supplier recommendations. The reference strain NCTC11168, was used as positive control in all assays, since the strain is known to contain the 40 assay genes.

PCR results were converted into binary data corresponding to the absence (0) or the presence (1) of the genetic marker in bacterial genomes and were stored into BioNumerics® software (v 6.5, Applied Maths, Belgium). A dendrogram was built using the simple matching distance coefficient and unweighted-pair group method using average linkages (UPGMA) of clustering in BioNumerics®, as previously described (Taboada et al., 2012). Clusters were defined on a basis of 90% of fingerprinting similarity (CGF40–90% clusters) to analyze the population structure. CGF40–90% clusters including at least 4% of the isolates of cattle or human population were defined as predominant in the population.

### Multilocus sequence typing (MLST)

Alleles of the seven housekeeping genes for MLST (*aspA, glnA, gltA, glyA, pgm, tkt*, and *uncA*) were determined within clinical isolates from 2009 in a previous study (Thépault et al., under review). In this study, alleles, sequence types (ST) and clonal complexes (CC) of isolates from cattle and clinical cases from 2015 were determined by Whole Genome Sequencing (WGS), using Ion Torrent technology available at the Anses sequencing platform, and by comparison of the sequences to the publically available PubMLST database (http://pubmlst.org/campylobacter) on BIGSdb (Jolley and Maiden, 2010). MLST characterization through WGS was performed on selected subsets of 77 and 79 isolates from cattle and clinical cases from 2015, respectively. Isolates selection was made to keep the same genetic structure in the subsets than in original populations, by selecting equal proportions of each CGF40 genotypes within cattle and clinical isolates from 2015, than proportions of these clusters observed in each original population. Clonal complexes including at least 4% of the isolates of cattle or human population were defined as predominant in the population.

### Data analysis

A chi-square test was used to assess the statistical difference between data collected from thermophilic *Campylobacter* prevalence analysis. The genetic diversity of *C. jejuni* within the different populations of isolates was assessed using Simpson's diversity index (ID) (Hunter and Gaston, 1988), calculated using the online tool “Comparing Partitions” from the website http://www.comparingpartitions.info (Carriço et al., 2006). The calculation of the confidence intervals for clustering agreement measures at 95% was performed using the resampling technique jackknife (Severiano et al., 2011).

## Results

### Prevalence of thermophilic *Campylobacter* spp.

In this study, the overall prevalence of thermophilic *Campylobacter* spp. in cattle was 69.1% (CI_95%_ = 66.1, 72.1), and a significant difference was observed between the contamination of adult cattle (39.3%; CI_95%_ = 34.9, 43.9) and calves (99.4%; CI_95%_ = 98.2, 99.9) with only three thermophilic *Campylobacter* negative calves (*p* < 0.001; Table 1). Within the adult cattle, the prevalence of thermophilic *Campylobacter* in dairy animals (46.9%; CI_95%_ = 40.1, 53.9) was significantly higher than among beef animals (33.3%; CI_95%_ = 27.8, 39.3) (*p* < 0.001; Table 1), while no significant difference was observed between dairy and beef calves.

The most common thermophilic *Campylobacter* species found in cattle of all ages was *C. jejuni* with a prevalence of 67.7% (CI_95%_ = 64.6, 70.6), followed by *C. coli* with a prevalence of 7.6% (CI_95%_ = 6.0, 9.5). Regarding *C. jejuni* prevalence according to cattle ages and breed, we observed in adult animals a prevalence of 37.3% with a significant difference (*p* < 0.001) between beef (31.5%) and dairy cattle (44.6%; Table 1). On another hand, within calves, the prevalence of *C. jejuni* was very high (98.5%; CI_95%_ = 96.9, 99.4) regardless of the breed (Table 1). Additional *Campylobacter* species were isolated from cattle to a lesser extent. Indeed, one dairy cattle was contaminated by *C. lari* and one beef cattle by *C. fetus*, while one beef calf carried *C. hyointestinalis* (Table 1). Interestingly, in almost 10% of animals, co-infections with several *Campylobacter* species were observed. Indeed, six adult cows and 54 calves were co-infected by *C. jejuni* and *C. coli*, while three different *Campylobacter* species were isolated from one calf (*C. jejuni, C. coli*, and *C. hyointestinalis*).

### CGF40

**Figure 1:**
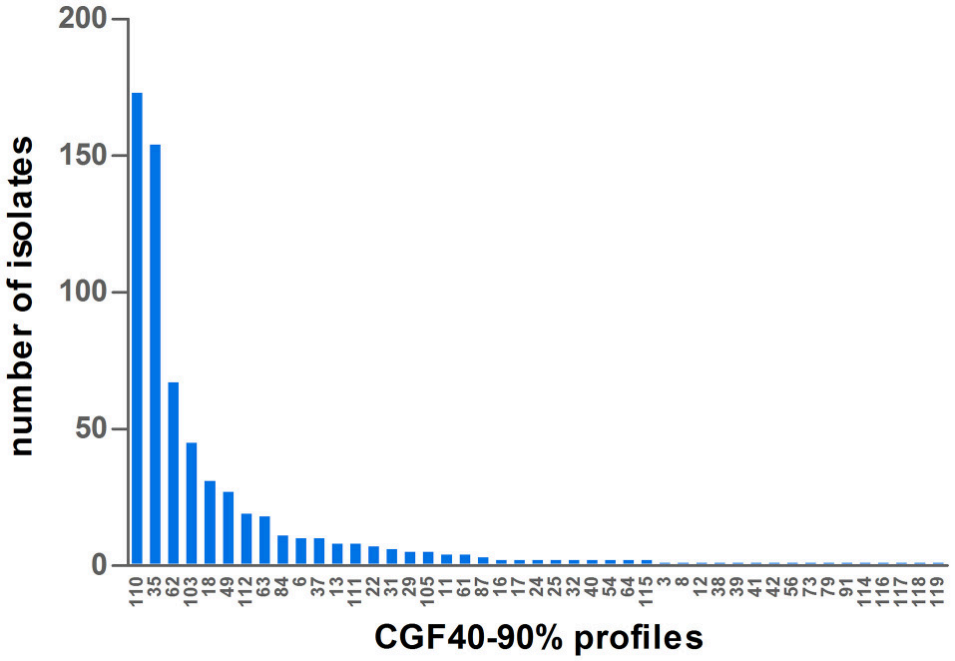
Distribution of CGF40–90% profiles within the population of 649 isolates of *C. jejuni* isolated from cattle in France.

**Figure 2:**
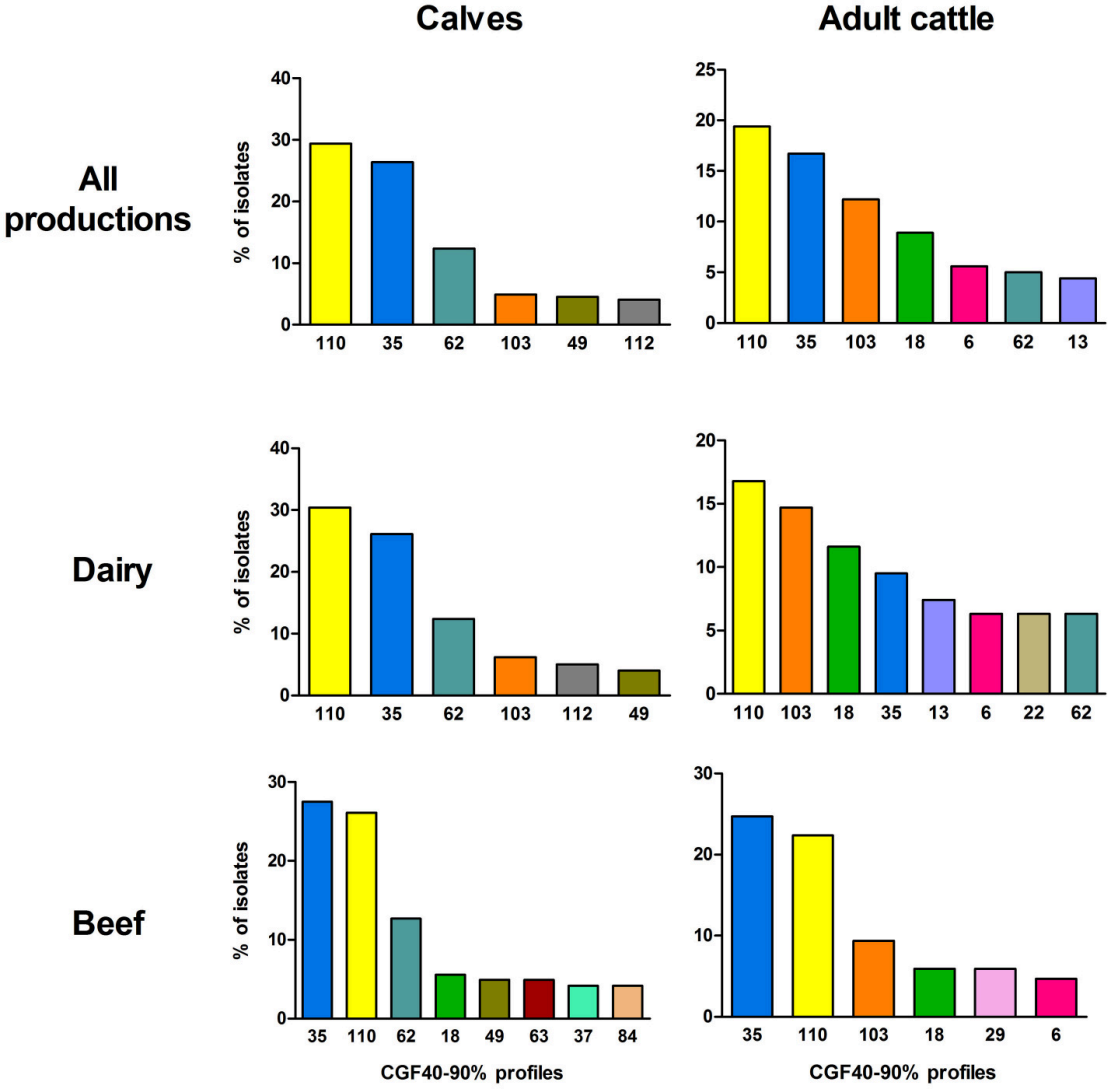
Prevalence of the most frequent CGF40–90% profiles (including ≥4% of isolates) within the different populations of *C. jejuni* isolates from calves and adults cattle.

CGF40–90% analysis revealed 45 distinct profiles among the 649 *C. jejuni* isolates collected in cattle and six clusters accounted for 76.58% of the isolates: the clusters 110, 35, 62, 103, 18, and 49 (Figure 1). Within calves isolates, which showed 29 different CGF40–90% clusters (Table 2), 6 clusters were predominant accounting for 81.66% of the isolates: the clusters 110, 35, 62, 103, 49, and 112 (Figure 2). These six clusters also predominated among dairy calves isolates, while only four (35, 110, 62, and 49) of these clusters were predominant within beef calves. Within beef calves, four supplementary main clusters were shown: clusters 18, 63, 37, and 84.

**Table 2:** Simpson's diversity indexes within the different populations of *C. jejuni* isolates.

***C. jejuni*** **population**		**Number of isolats**	**Number of partitions**	**Simpson index (CI_95%_)^b^**
Cattle		649	**45**	**0.851 (0.834–0.868)**
	Adult cattle	180	29	0.904 (0.883–0.924)
	*Dairy*	*95*	*20*	*0.915 (0.894–0.937)*
	*Beef*	*85*	*22*	*0.876 (0.833–0.918)*
	Calves	469	29	0.820 (0.799–0.842)
	*Dairy*^a^	*322*	*25*	*0.816 (0.790–0.842)*
	*Beef*^a^	*142*	*16*	*0.833 (0.798–0.869)*
Human		514	**98**	**0.952 (0.944–0.960)**

a *Two mixed breed calves and 3 calves with an unknown breed have been removed from this table*.

b *The Simpson's diversity indexes reflect the genetic diversity within a population of individuals*.

Then, with regards to the adult cattle isolates population, which showed 29 CGF40–90% clusters (Table 2), seven main clusters gathered 72.22% of the isolates: the clusters 110, 35, 103, 18, 6, 62, and 13 (Figure 2). Only four main clusters (110, 35, 62, and 103) were common to adult cattle and calves (Figure 2). In the same way than within the calves, the distribution of the main clusters between dairy and beef breed was different. Among dairy cattle, eight main clusters (110, 103, 18, 35, 13, 6, 22, and 62) gathered 78.94% of the isolates while within beef cattle, only six predominant clusters (35, 110, 103, 18, 29, and 6) grouped 72.94% of the isolates.

According to the Simpson's diversity indexes and their confidence intervals (CI_95%_), *C. jejuni* isolates collected from adult cattle showed a higher significant genetic diversity than isolates from calves as no overlap was observed between their CI_95%_ (Table 2). On the contrary, no significant difference of genetic diversity was observed between isolates collected from different cattle breeds (dairy vs. beef).

**Figure 3:**
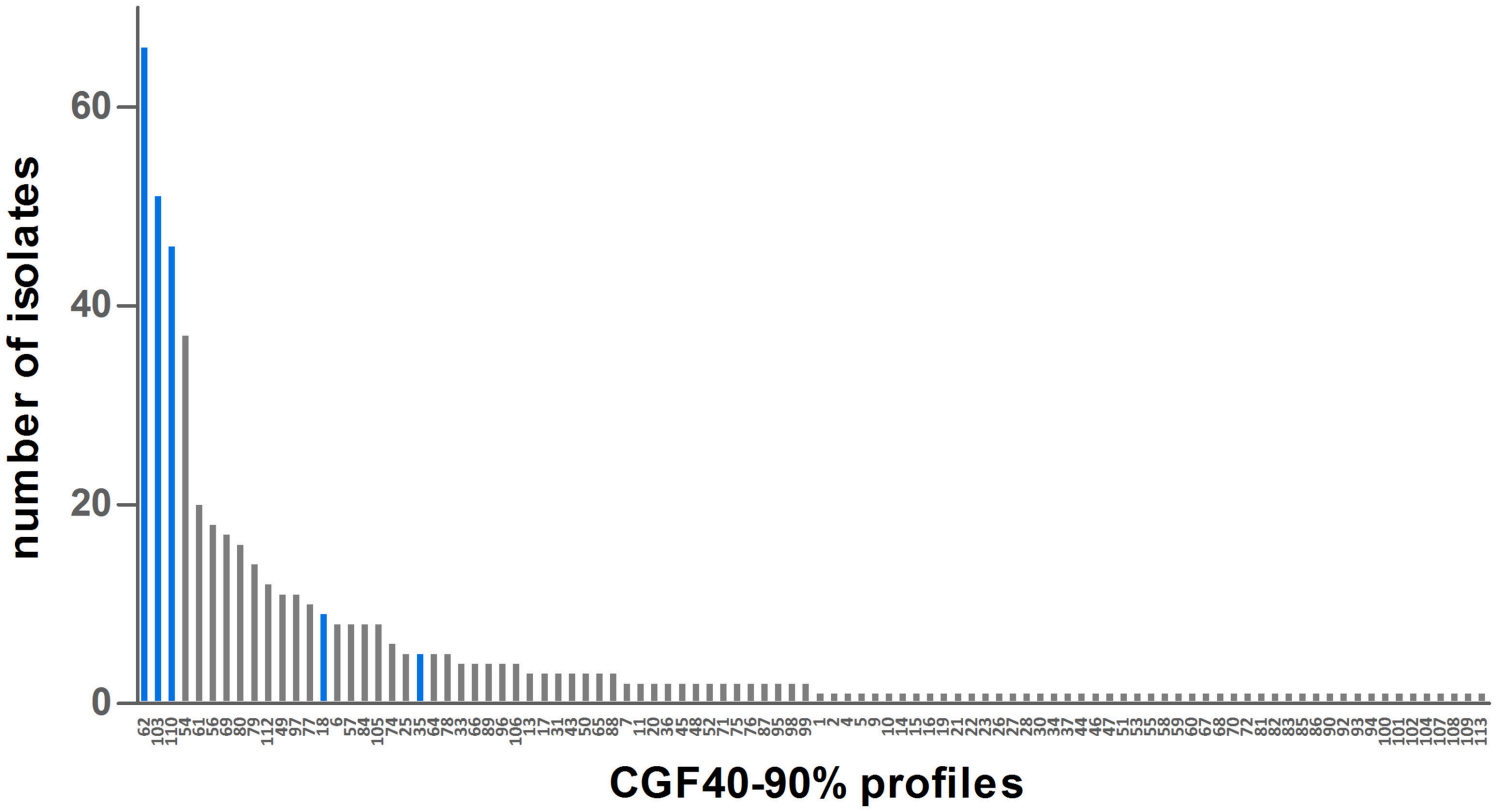
Distribution of CGF40–90% profiles within the population of 514 clinical isolates from France. Blue bars represent CGF40–90% clusters which are the most frequently found in cattle isolates.

Nevertheless, genetic diversity of cattle *C. jejuni* isolates was significantly lower than that observed among clinical isolates (Table 2). Indeed, 98 different CGF40–90% profiles were shown among the 514 clinical isolates from 2009 and 2015 typed. However, the genetic diversity in clinical isolates seems to be stable overtime since the main clinical clusters (clusters 62, 103, 110, and 54) observed in 2009 (55.2%) and 2015 (32.6%) are identical. These four main profiles gathered 38.9% of all clinical isolates, (Figure 3) and 49 of the 98 clusters appeared only once in the population (unique clusters). Interestingly, among the four main prevalent clusters within the clinical isolates, 3 were also predominant within the cattle isolates: the clusters 62, 103, and 110. Moreover, the 3 other main clusters within the cattle isolates, clusters 49, 18, and 35, were also shown by 11, 9, and 5 clinical isolates respectively (Figure 3).

### MLST

From the selection of 77 cattle isolates, 26 STs were identified, of which 14 appeared only once in the data set. Except ST-2217, ST-5707, ST-8968, ST-8967, and ST-8969, each represented by a single isolate, all STs identified were grouped into 9 clonal complexes, with more than 80% of isolates belonging to the four most prevalent lineages (CC-21, CC-61, CC-48, and CC-257, Table 3). From the 222 clinical isolates typed (including isolates from 2009 and isolates from 2015), 94 STs were identified, of which 58 appeared only once in the data set and more than 93% of isolates were grouped into 24 clonal complexes (Table 3). The 6 main clonal complexes (CC-21, CC-206, CC-48, CC-353, CC-354, and CC-257) gathered almost 65% of the isolates (Table 3).

**Table 3:** Clonal complexes found among the cattle and clinical isolates and link with the CGF40–90% clusters.

**Cattle isolates**			**Human isolates**								
**Clonal complex**	**CGF40-90% cluster**	**Numberofisolates**	**Clonal complex**	**CGF40-90% cluster**	**Number of isolates**	**Clonal complex**	**CGF40-90% cluster**	**Numberofisolates**	**Clonalcomplex**	**CGF40-90% cluster**	**Number of isolates**
ST-21 complex		33	ST-21 complex		72	ST-257 complex		10	ST-52 complex		4
	61	1		54	1		**49**	2		56	3
	63	1		61	6		50	1		97	1
	**103**	9		**62**	1		66	1	ST-61 complex		3
	105	1		89	1		74	5		35	2
	**110**	20		100	1		79	1		**62**	1
	112	1		101	1	ST-45 complex		7	ST-574 complex		3
ST-61 complex		16		**103**	25		7	2		56	2
	**35**	16		105	3		11	1		89	1
ST-48 complex		8		106	3		13	2	ST-403 complex		3
	**62**	6		**110**	24		16	1		31	3
	63	2		112	6		88	1	ST-952 complex		2
ST-257 complex		5	ST-206 complex		20	ST-464 complex		8		48	2
	**49**	5		61	3		1	1	ST-607 complex		2
ST-42 complex		3		**62**	15		80	7		54	1
	**18**	3		69	2	ST-22 complex		6		61	1
ST-45 complex		3	ST-48 complex		16		15	1	ST-362 complex		2
	6	1		56	1		25	4		**18**	2
	11	1		**62**	13		**62**	1	ST-446 complex		1
	13	1		65	1	ST-443 complex		6		58	1
ST-206 complex		2		69	1		54	1	ST-460 complex		1
	62	2	ST-353 complex		15		57	5		66	1
ST-283 complex		1		54	2	ST-283 complex		5	ST-49 complex		1
	6	1		**62**	1		6	3		64	1
ST-658 complex		1		77	6		**62**	1	ST-677 complex		1
	84	1		78	2		**110**	1		17	1
No complex		5		79	4	ST-658 complex		5	No complex		14
ST-2217	18	1	ST-354 complex		11		54	2	ST-5707	**18**	1
ST-5707	29	1		54	10		84	2	ST-3578	26	1
ST-8967	62	1		97	1		96	1	ST-534	27	1
ST-8968	103	1				ST-42 complex		4	ST-905/ST-8970	33	2
ST-8969	110	1					**18**	3	ST-8971	43	1
							22	1	ST-1374 (1)/ST-2274 (2)	54	3
									ST-4833	60	1
									ST-2258	65	1
									ST-1399	71	1
									ST-441	87	2

*Bold values correspond to the main CGF40-90% clusters observed in cattle isolates*.

## Discussion

In this large scale study designed to be representative for the cattle production in France, we assessed the contamination of cattle by thermophilic *Campylobacter* spp. and described the genetic structure of *C. jejuni* isolates circulating in these animals using CGF40 and MLST. Here, we reported a high contamination of cattle by thermophilic *Campylobacter* spp. (69.1%), with the predominance of *C. jejuni* which showed a prevalence of 67.7%. *C. jejuni* is the main species reported in cattle (Kwan et al., 2008; Fernandez and Hitschfeld, 2009), but wide range of prevalence estimates were described. They can vary for *Campylobacter* spp. from 16.5 to 89.4% according to the study (Stanley et al., 1998; Hakkinen et al., 2007; Kwan et al., 2008; Fernandez and Hitschfeld, 2009; Rotariu et al., 2009; Châtre et al., 2010). Surprisingly, the comparison of prevalence estimates obtained in the present study with results from a previous work performed on cattle in France (Châtre et al., 2010), revealed significant discrepancies between prevalence estimates (69.1 vs. 16.5% for thermophilic *Campylobacter* spp.). One of the most likely hypotheses to explain the discrepancies is the differences in protocols used for the detection of thermophilic *Campylobacter*, and especially the absence of an enrichment step for the isolation of thermophilic *Campylobacter* in Châtre et al. (2010) work. Thus, if we consider only direct platting results in our study, the thermophilic *Campylobacter* prevalence would have been 86.3% in adult cattle and 13.7% in calves, instead of 99.4 and 39.3%, respectively. Indeed, since the contamination level of cattle by *Campylobacter* is thought to be generally low (10^2^ to 10^4^ CFU/g) compared with chicken (Nielsen, 2002; Hakkinen et al., 2007; Rotariu et al., 2009), and since a significant microbial flora can be observed on agar plate with cattle samples (Workman et al., 2005), less isolation of *Campylobacter* can occur from the most weakly contaminated animals without enrichment step. On another hand, it has been reported that the search of *Campylobacter* in intestinal samples generally gave higher estimates than study with feces (Stanley and Jones, 2003).

In addition to technical differences in protocols experiment, several other factors may influence prevalence estimates, such as the size and type (breed) of herd, the season, animal ages, geography, or husbandry practices (Stanley and Jones, 2003). The impact of animal age on *Campylobacter* prevalence has been widely described (Nielsen, 2002; Johnsen et al., 2006; Châtre et al., 2010) and our results emphasize this finding. In the present work, thermophilic *Campylobacter* prevalence in calves was shown to be significantly higher than adult cattle with 99 and 39% of positive animals respectively. Several hypotheses may explain this difference, such as more frequent exposure to *Campylobacter* in calves, or the acquired immunity in adult animals which may prevent infection (Johnsen et al., 2006). In this study, we also highlighted differences in prevalence estimates according to the type of herd. Indeed, a significantly higher contamination of dairy adult cattle compared with beef adult cattle was observed, while this difference was not observed between calves. However, a previous study highlighting differences according to the herd type, showed the opposite result with a higher prevalence rate in beef cattle compared with dairy animals (Fernandez and Hitschfeld, 2009). Fernandez and Hitschfeld (2009) suggested that this higher prevalence in beef cattle was due to husbandry conditions of animals with a higher exposition to the environment in beef animals. Then, seasonal variations are also described in some studies (Stanley et al., 1998; Hakkinen and Hanninen, 2009).

Here, we thus identified cattle as an important reservoir for thermophilic *Campylobacter* and for *C. jejuni* especially. Genotyping using CGF40–90% of *C. jejuni* isolates highlighted the existence of 45 clusters within 649 isolates, in which six profiles were most prevalent accounting for 76.58% of the overall cattle isolates population. Some of these six most prevalent profiles were frequently found in a large proportion in the different populations of cattle isolates (dairy or beef calves and dairy or beef adult cattle). Nevertheless, each population of isolates showed in addition their own most prevalent profiles (e.g., clusters 63, 37, or 84 in beef calves). Genotyping of *C. jejuni* isolates from cattle showed the CC-21, CC-61, CC-48, and CC-257 as the most common CCs found in these animals, and are in accordance with a previous study which also described CC-61 or CC-21 as predominant in cattle (Kwan et al., 2008; Grove-White et al., 2011). Based on CGF40–90% clusters, Simpson's diversity index calculated for each *C. jejuni* population of isolates from cattle did not show any differences in genetic diversity with the exception of adult cattle and calves. Indeed, *C. jejuni* from calves showed significantly lower genetic diversity than *C. jejuni* from adults. When compared to clinical isolates, cattle isolates showed a significantly lower genetic diversity.

We genotyped in this study isolates from clinical cases to assess the potential link between cattle and clinical isolates. A significant genotypes overlap was found between clinical and cattle isolates, since 3 of the 4 main clinical CGF40–90% clusters were predominant in cattle isolates, suggesting a significant implication of cattle in clinical cases. However, this observation needs to be nuanced, since with regards to MLST genotypes, 3 of the 4 main CCs found in common between cattle and clinical cases (CC-21, CC-48, CC-257) are frequently described in other hosts (Sheppard et al., 2014; Guyard-Nicodème et al., 2015; Thépault et al., under review). Indeed, CC-21 and CC-48 are defined as host generalist clonal complexes, as they are frequently isolated from various reservoirs (including poultry and pets), while the CC-257 is described as chicken specialist (Sheppard et al., 2014). Therefore, no direct link can be made between these cattle and clinical isolates.

In conclusion, this large scale survey allowed the identification of cattle as a significant reservoir for thermophilic *Campylobacter*, and especially for *C. jejuni*. Calves appeared to be significantly more contaminated than adults, as well as dairy cattle compared with beef animals. Genotyping of isolates highlighted the lower genetic diversity of cattle isolates compared with clinical *C. jejuni* and enabled the first insights in the description of the genetic structure of *C. jejuni* in cattle. Clonal complexes found in cattle in France are consistent with previously described, but included for the most part of them host generalist clonal complexes. The significant overlap between genotypes found in cattle and clinical isolates from 2009 and 2015 suggest a potential important implication of this animal reservoir in human campylobacteriosis.

## Author contributions

AT, KR, and MC contributed to the conception and design of the work, carried out sampling, and bacteriological analysis, analyzed data, and wrote the paper. TP and SQ carried out sampling and bacteriological analysis. SQ, VR, and AT performed typing experiments.

### Conflict of interest statement

The authors declare that the research was conducted in the absence of any commercial or financial relationships that could be construed as a potential conflict of interest.
